# The correlation with tumor radiological characteristics and prognosis of patients with early-stage hepatocellular carcinoma receiving transcatheter arterial chemoembolization

**DOI:** 10.1097/MD.0000000000026414

**Published:** 2021-07-02

**Authors:** Shou-Wu Lee, Teng-Yu Lee, Yu-Chi Cheng, Chieh-Ling Yen, Sheng Shun Yang

**Affiliations:** a Division of Hepatology and Gastroenterology, Department of Internal Medicine, Taichung Veterans General Hospital; b Department of Internal Medicine, Chung Shan Medical University, Taichung; c Department of Radiology, Taipei Veterans General Hospital; d Department of Internal Medicine, Yang Ming University, Taipei, Taiwan.

**Keywords:** Hepatocellular carcinoma, radiological characteristics, transcatheter arterial chemoembolization

## Abstract

Transcatheter arterial chemoembolization (TACE) is a standard treatment modality for intermediate stage hepatocellular carcinoma (HCC). The aim of this study was to determine the tumor radiological characteristics associated with prognosis of patients with early-staged HCC receiving TACE.

Patients with BCLC stage A HCC were enrolled at Taichung Veterans General Hospital from January 2005 to December 2009. According to mRECIST criteria, patients with or without complete response (CR) were assigned to either the CR group or the non-CR group. Clinical and radiological characteristics were compared between the two groups.

In 40 enrolled patients, 24 (60%) were in the CR group, and 16 (40%) in the non-CR group. Clinical parameters were similar between the 2 groups. Radiological characteristics including complete lipidol retention and the absence of residual tumor blush were significantly correlated with achievement of CR in the patients with small-sized HCC. Patients with CR after TACE had a significantly longer overall survival than those without (31.29 vs 22.63 months, *P* = .021).

Complete lipidol retention and the absence of residual tumor blush were correlated with the radiological complete tumor response of these patients with small-sized early-stage HCC receiving TACE.

## 1 Introduction

Hepatocellular carcinoma (HCC) is the most common cancer worldwide, and it predominantly develops in patients with liver cirrhosis.^[1]^ HCC occurs most often in the setting of underlying chronic liver diseases such as advanced fibrosis or cirrhosis, that is typically related to infections with hepatitis B virus (HBV), hepatitis C virus (HCV), or with prolonged alcohol or nonalcoholic fatty liver disease.^[2]^ The Barcelona Clinic Liver Cancer (BCLC) staging system integrates tumor characteristics and performance status with liver function, and these features are linked to evidence-based therapeutic options.^[3]^ For BCLC stage A, or early stage HCC, the recommended treatment modalities are curative treatments such as hepatic resection, liver transplantation, and radiofrequency ablation (RFA). However, these modalities sometimes cannot be applied to all the patients with HCC due to the individual conditions.

Transcatheter arterial chemoembolization (TACE) is the recommended treatment modality for BCLC stage B, or intermediate stage HCC.^[4]^ In patients with unresctable HCC, TACE can slowdown cancer progression and thereby improving their survivals, compared with those treated with the best supportive care.^[5,6]^ In addition, some clinical studies have evaluated patients with solitary HCC undergoing TACE.^[7,8]^ However, therapeutic outcomes of these patients remain variable in the literature.

The aim of our study was to determine the relationship between tumor radiological characteristics and the prognosis of patients with early-stage HCC receiving TACE as their primary treatment.

## 2 Methods

The newly diagnosed HCC patients in accordance with AASLD guideline^[9]^ from January 2005 to December 2009 at Taichung Veterans General Hospital were collected. Criteria of enrollment included those in BCLC stage A HCC under primary treatment with TACE. These subjects were considered not eligible for hepatic resection, liver transplantation or RFA due to multiple comorbidities, no suitable donor, and risks of postoperative hepatic dysfunction or tumors in locations difficulty to approach. Exclusion criteria included those diagnosed with tumor major vascular invasion or extrahepatic spread, cirrhotic Child-Pugh stage C, displayed a poor performance status, or loss of follow-up within the following day.

Clinical parameters of the patients, including age, gender, liver function such as total bilirubin, alanine aminotransferase (ALT), alpha-fetoprotein (AFP), presence of chronic HBV and HCV infection, cirrhotic Child-Pugh stage, and the tumor size were collected.

The study project is approved by the Institutional Review Board of Taichung Veterans General Hospital (104DHA0500198).

### 2.1 TACE technique

TACE was performed under patients’ written informed consent. A 5 or 6-French sheath was inserted into the common femoral artery. Digital subtraction was first performed on angiograms of the celiac and superior mesenteric arteries to assess the portal vein patency, vascular anatomy, and tumor vascularity. All angiographic images were stored in an image archive and communication system. Following the initial arterial assessment, the catheter was advanced into the lobar or segmental hepatic artery supplying the tumor. If the initial 4-Fr or 5-Fr diagnostic catheter had been successfully advanced into the optimal position, it was used for the TACE infusion. In other cases when more selective catheterization was required, a 2.9-Fr microcatheter of Progreat (Terumo, Tokyo, Japan) was used instead. In general, the TACE infusion site was chosen to enable selective tumor embolization. In the event of encountering separate arterial feeders, or a discrete blood supply was not identified, a right or left hepatic arterial infusion was performed. When the hepatic synthetic function was a concern, a selective approach was chosen. Once the lesion and its blood supply had been identified, under fluoroscopic guidance an emulsion of 10 to 50 mg epirubicin (Pfizer, NY) and 2 to 60 mL Lipidol (Guerbet, Aulnay sous Bois, France) was injected into the arterial supply of the tumor. For patients with liver or renal dysfunction, leukopenia, and thrombocytopenia, their doses of chemotherapy agents were adjusted accordingly. Administration of the emulsion was followed by embolization with a slurry of Spongostan (Ethicon, NJ) until stasis had been achieved.

### 2.2 Radiological characteristics

For superselective TACE, the tip of the catheter was placed into the hepatic arterial branch afferent to the segment or subsegment sites where the tumor was located. Their radiological findings were recorded. In nonselective TACE, a lobar technique was carried out in the case of a nodule fed by multiple arteries. On the post-TACE angiogram, tumor-feeding artery blockage was defined according to whether or not tumor-supplying arteries were visualized. Presence of residual tumor blush was defined as either an unchanged tumor stain or a reduction in intensity or size compared with the pre-TACE image. According to post-TACE CT images, depending on the pattern of tumor covered by lipidol, complete lipidol retention was defined as >90% lipidol retention without peripheral filling defects.

### 2.3 Assessment of responses following TACE

Patients were assessed every 2 months by dynamic imaging study until reaching the endpoints, including death, disease progression, or treatment failure after TACE. The best tumor response was assessed according to the modified RECIST (mRECIST) criteria^[10]^ with 4 response categories: complete response (CR), partial response (PR), stable disease (SD), and progressive disease (PD). Patients with CR were included in the complete response (CR) group, while all remaining patients were included in the non-complete response (non-CR) group.

### 2.4 Statistical analyses

Data were expressed as mean and standard deviation for each of the measured parameters. The positive rate of each group was both expressed as a percentage of the total numbers of patients. Statistical comparisons across groups were made using Pearson Chi-square test or Fisher exact test. Continuous variables were analyzed with the independent *t* test. Statistically significances were set at *P* value < .05. Hazard ratios (HRs) with a 95% confidence interval (95% CI) were calculated by univariate and multivariate Cox's regression to examine the strength of associations between the clinical or radiological variables and the responses following TACE. Survival analysis was carried out using the Kaplan–Meier method for analysis.

## 3 Results

A total of 40 patients were enrolled, and their characteristics and outcomes are summarized in Table 1. Their median age was 69.58 years, with more males (77.5%). Chronic infections of HBV were found in 12 patients (30.0%), and of HCV in 19 patients (47.5%). Child-Pugh stages A occurred in 28 patients (70.0%) and stage B in 12 patients (30.0%). Most patients (95%, 38 cases) had solitary HCC. The median HCC diameter was 2.73 cm, with 57.5% (n = 23 cases) having tumor size ≤3 cm and 42.5% (n = 17 cases) >3 cm.

**Table 1:** The general data and outcomes of the patients with TACE.

	All (N = 40)		
Table 1	M ± SD	N	%
Age, yr	69.58 ± 12.60		
Gender (male)		31	(77.5%)
Viral hepatitis			
HBV		12	(30.0%)
HCV		19	(47.5%)
HBV/HCV		4	(10.0%)
nil		5	(12.5%)
Cirrhosis, Child-Pugh			
A		28	(70.0%)
B		12	(30.0%)
Total bilirubin, mg/dL	1.23 ± 0.70		
ALT, u/L	59.73 ± 44.11		
AFP, ng/mL	184.54 ± 403.71		
HCC numbers			
1		38	(95.0%)
2–3		2	(5%)
HCC size, cm	2.73 ± 1.11		
≤ 3 cm		23	(57.5%)
> 3 cm		17	(42.5%)
Radiological responses			
CR		24	(60.0%)
PR		12	(30.0%)
SD		2	(5.0%)
PD		2	(5.0%)
Non-CR (PR+SD+PD)		16	(40.0%)
ORR (CR+PR)		36	(90.0%)
DCR (CR+PR+SD)		38	(95.0%)
Overall survival, mo	27.83 ± 10.91		

AFP = alpha-fetoprotein, ALT = alanine aminotransferase, CR = complete response, DCR = disease control rate, HBV = hepatitis B, HCC = hepatocellular carcinoma, HCV = hepatitis C, M = mean, N = number of patients, OR = objective response, ORR = objective response rate, PD = progressive disease, PR = partial response, SD = stable disease, SD = standard derivation.

The numbers of cases with CR, PR, SD, and PD were 24 (60.0%), 12 (30.0%), 2 (5.0%), and 5, (5.0%) respectively. Among those receiving TACE, the objective response rate (ORR) was 90.0%, and the disease control rate (DCR) was 95.0%. Overall, among these patients, 60.0% (n = 24) were in the CR group and 40.0% (n = 16) in the non-CR group. The average numbers of TACE in the CR group to achieve complete tumor response were 1.33 ± 0.70 (once in 19 cases, twice in 2 cases, and thrice in 3 cases). The overall survival of all patients was 27.83 ± 10.91 months.

Patient characteristics and radiological characteristics of both the CR and non-CR groups are summarized in Table 2. No intergroup differences were found regarding age, gender, rate of chronic viral hepatitis infection, Child-Pugh stage, and laboratory parameters, including total bilirubin, ALT, and AFP. Those patients with smaller (≤3 cm) HCC had a higher rate of CR than those with larger (>3 cm) HCC did (69.6% vs 47.1%), but the difference was not significant (*P* = .151) The CR group had a significant higher rate of no residual tumor blush (100% vs 75.0%, *P* = .020), compared with the non-CR group. Although rates of super-selection for TACE (62.5% vs 81.3%, *P* = .297) showed no differences, rates of complete lipidol retention were also similar (87.5% vs 68.8%, *P* = .229).

**Table 2:** The general data and radiological characteristics of the non-CR group and the CR group.

	Non-CR (N = 16, 40%)			CR (N = 24, 60.0%)			
Table 2	M ± SD	N	%	M ± SD	N	%	*P*
Age, yr	70.25±12.18			69.13±13.12			.786^∗^
Gender (male)		11	(68.8%)		20	(83.3%)	.242^‡^
Viral hepatitis							.189^‡^
HBV		5	(31.2%)		7	(29.1%)	
HCV		10	(62.5%)		9	(37.5%)	
HBV/HCV		0			4	(16.7%)	
nil		1	(6.3%)		4	(16.7%)	
Cirrhosis, Child-Pugh							.398^†^
A		10	(62.5%)		18	(75.0%)	
B		6	(37.5%)		6	(25.0%)	
Total bilirubin, mg/dL	1.31 ± 0.79			1.17 ± 0.64			.524^∗^
ALT, u/L	72.00 ± 51.87			51.54 ± 37.02			.183^∗^
AFP, ng/mL	126.54 ± 275.44			223.20 ± 472.21			.465^∗^
HCC numbers							1.000^‡^
1		15	(93.8%)		23	(95.8%)	
2–3		1	(6.2%)		1	(4.2%)	
HCC size, cm	3.06 ± 1.12			2.50 ± 1.06			.117^∗^
≤ 3 cm		7	(30.4%)		16	(69.6%)	.151^†^
> 3 cm		9	(52.9%)		8	(47.1%)	
Radiological characteristics							
Super-selective TACE		13	(81.3%)		15	(62.5%)	.297^‡^
Complete lipidol retention		11	(68.8%)		21	(87.5%)	.229^‡^
Tumor feeding artery blockage		13	(81.3%)		17	(70.8%)	.456^‡^
Absence of residual tumor blush		12	(75.0%)		24	(100%)	.020^‡^

AFP = alpha-fetoprotein, ALT = alanine aminotransferase, CR = complete response, HBV = hepatitis B, HCC = hepatocellular carcinoma, HCV = hepatitis C, M = mean, N = number of patients, SD = standard derivation.

∗ *P*-values were analyzed with independent *t* test.

† Pearson Chi-square test.

‡ Fisher exact test.

Logistic analysis of individual items to appearance of complete tumor response following TACE is summarized in Table 3. Absence of residual tumor blush had a positive impact on achievement of CR after TACE according to univariate analyses (HR 12.84, 95% CI 2.86–61.27, *P* = .012), but the strength of this association became nonsignificant after adjusted with multivariate analyses (HR 4.01, 95% CI 0.82–12.60, *P* = .724).

**Table 3:** The strength of associations between the variables and complete tumor response following TACE.

	Univariable analysis			Multivariable analysis		
Table 3	HR	(95% CI)	*P*	HR	(95% CI)	*P*
Age	1.01	(0.96–1.04)	.922			
Gender (male vs female)	1.44	(0.34–6.08)	.616			
Viral hepatitis (HBV vs HCV)	0.55	(0.05–6.26)	.427			
Cirrhosis, Child-Pugh stage (A vs B)	1.36	(0.36–5.13)	.646			
AFP	1.00	(0.99–1.01)	.979			
HCC numbers (1 vs 2–3)	1.80	(0.41–7.91)	.436			
HCC size (≤3 vs >3 cm)	2.45	(0.59–10.19)	.216			
Super-selective TACE	0.73	(0.19–2.85)	.654			
Complete lipidol retention	1.97	(0.50–7.83)	.336			
Tumor feeding artery blockage	0.59	(0.14–2.57)	.482			
Absence of residual tumor blush	12.84	(2.86–61.27)	.012	4.01	(0.82– 12.60)	.724

AFP = alpha-fetoprotein, CI = confidence interval, HBV = hepatitis B virus, HCC = hepatocellular carcinoma, HCV = hepatitis C virus, HR = hazard ratio, N = number of patients, TACE = transcatheter arterial chemoembolization.

Table 4 summarizes the further analysis of radiological characteristics based on subclassification of HCC size (≤3 or >3 cm). For subjects with ≤3 cm HCC, their CR cases, compared with the non-OR cases, showed a significantly higher rate of complete lipidol retention (87.5% vs 42.9%, *P* = .045) and more cases with absence of residual tumor blush (100% vs 57.1%, *P* = .020). However, no such differences were found for to the subjects with >3 cm HCC.

**Table 4:** The radiological characteristics of the small and large-sized HCC.

	Non-CR		CR		
	N	%	N	%	*P*
HCC ≤ 3 cm	7		16		
Super-selection for TACE	6	(85.7%)	11	(68.8%)	.621
Complete lipidol retention	3	(42.9%)	14	(87.5%)	.045
Tumor feeding artery blockage	6	(85.7%)	14	(87.5%)	1.000
Absence of residual tumor blush	4	(57.1%)	16	(100%)	.020
HCC > 3 cm	9		8		
Super-selection for TACE	7	(77.8%)	4	(50.0%)	.335
Complete lipidol retention	8	(88.9%)	7	(87.5%)	1.000
Tumor feeding artery blockage	7	(77.8%)	3	(37.5%)	.153
Absence of residual tumor blush	8	(88.9%)	8	(100%)	1.000

*P*-values were analyzed with Fisher exact test. CR = complete response, HCC = hepatocellular carcinoma, N = number of patients, TACE = transcatheter arterial chemoembolization.

**Figure 1:**
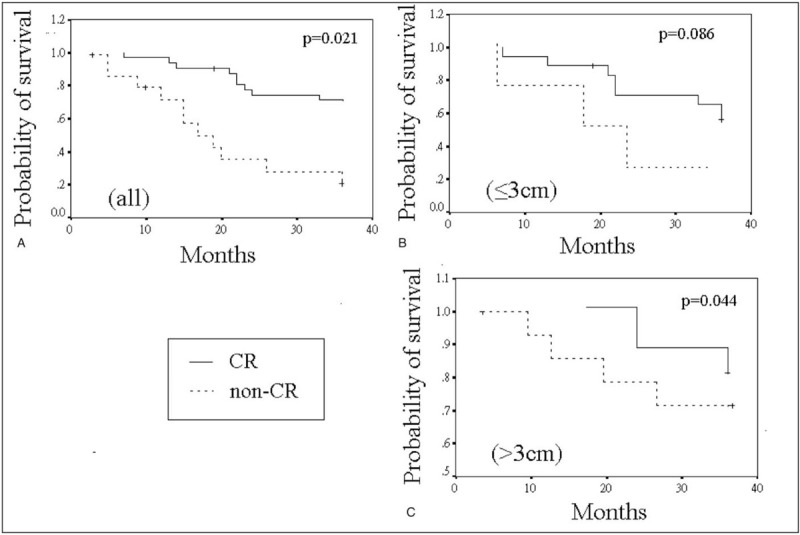
Comparison of overall survival between the non-CR group and the CR group. CR = complete response.

The outcomes of overall survival of the enrolled patients are shown in Figure 1. The CR group had a significantly longer overall survival compared with the non-CR group (31.29 ± 8.60 vs 22.63 ± 12.15 months, *P* = .021). The highest overall survival was found in those patients with CR and with >3 cm tumors (34.50 ± 4.24 months), following by those with CR and ≤3 cm tumors (29.68 ± 9.84 months), those with non-CR and >3 cm tumors (23.67 ± 13.29 months) and those with non-CR and ≤3 cm tumors (21.28 ± 11.40 months). No significant difference was found between cases with ≤3 cm tumors in the CR group and those in the non-CR group (*P* = .086). However, for patients with >3 cm tumors, achievement of CR with TACE showed a longer overall survival than those without achievement (*P* = .044).

## 4 Discussion

HCC is a common type of primary liver malignancy worldwide with about 30% to 40% of patients are diagnosed at an early stage.^[11]^ Recommended treatment modalities for resectable HCC are curative treatments, including hepatic resection, liver transplantation, and RFA. The treatment modalities for unresectable HCC are palliative treatments including TACE or systemic therapy.^[3]^ However, some patients with early-stage HCC are not suitable for these curative treatments, including those with old age, poor hepatic function, multiple comorbidities, lack of suitable donor, larger HCC size, or the tumor in locations difficult to approach. Alternatively, TACE was a treatment choice for such patients based on clinical judgment.

Radiological response assessment plays a central role in the evaluation of treatment success following TACE. The modified RECIST retains the concept of measuring the viable part of residual tumor tissue, but recommends the uni-dimensional assessment of the longest viable tumor diameter and the numeric definitions of response according to RECIST.^[10]^ Determination of objective treatment response following TACE by measuring residual viable tumor tissue is a surrogate marker of overall survival.^[12,13]^

In this study, we found the radiological CR in 24 of 40 patients (60%). Most clinical parameters, including age, gender, rates of chronic viral hepatitis infection, Child-Pugh stage, tumor numbers, and laboratory parameters, had no impact on their CR. On the contrary, patients with smaller tumors (≤3 cm) had a higher rate of CR than those with larger tumors (>3 cm) (69.6% vs 47.1%), although the difference was not statistically significant (*P* = .151).

One Italian study on 148 patients with solitary HCC undergoing TACE reported a 64% CR rate, and the small tumor being a significant predictor to achieve radiological CR (≤3 cm HCC vs >3 cm HCC: 73% vs 54%, *P* = .017).^[7]^ Another Korean study on 175 patients with solitary HCC receiving TACE reported a 68% CR rate and small tumors (≤3 cm) also being a significant predictor by multivariate analysis (odds ratio 2.024, 95% CI 1.295–6.135, *P* = .049).^[8]^ The reason of our not observing significant difference of CR rate between patients with large- and small-sized HCC might be due to our smaller sample size (n = 40) and smaller average tumor size (2.73 cm).

TACE involves the intra-arterial infusion of a cytotoxic drug, such as doxorubicin or cisplatin, that is emulsified in the oil-based radio-opaque agent lipidol. This is followed by embolization of the blood vessel using gelatin sponge particles or microspheres, resulting in a strong and sustained cytotoxic effect on top of ischemia.^[1]^ Therefore, the presence of a significant arterioportal shunt secondary to large-sized HCC may interfere with TACE, because anticancer drugs and iodized oils can easily pass through the shunt.

The tumor retention of lipidol is associated with tumor response and is considered a prognostic marker.^[14]^ A heterogeneous and incomplete lipidol pattern is correlated with a higher risk of recurrence,^[15,16]^ and the presence of lipidol in at least 75% of the lesion is a predictor of better survivals.^[17]^ Incomplete lipidol deposition appearance may be due to incomplete catheterization and treatment injection in all tumor feeders, resulting in lipidol particles being unable to penetrate the smallest tumor capillary vessels in the tumor.

In our study, two radiological characteristics, that is, complete lipidol retention and absence of residual tumor blush are significant predictors for achieving radiological CR after TACE in patients only with HCC sizes ≤3 cm, but not with sizes >3 cm. This discrepancy might be due to more non-well enhanced daughter nodules or satellite lesions around large-sized HCC, rendering it difficult to achieve CR with TACE.^[18]^

An objective radiological tumor response with TACE is an independent prognostic factor of overall survival.^[12,19]^ An earlier Italian study reported the average overall survival being 37 and 28 months in the patients with CR and non-CR after TACE, with a significant difference (*P* = .048).^[7]^ Our study also found a significant better overall survival in the CR group than that in the non-CR group (31.29 ± 8.60 vs 22.63 ± 12.15 months, *P* = .021), without an impact of tumor size on predicting the overall survival.

Our study has a few limitations. First, the study design was retrospective, likely with selection or reporting biases. Second, dosage of epirubicin and lipidol used in TACE were not recorded, and the dose-tumor response relationship was not determined. Third, patients had variable tolerance to TACE and the endpoints were operator-dependent. Fourth, subsequent alternative or combined tumor therapies were not considered, and tumor recurrences after radiological CR was also not recorded. Besides, the survivals of our cases following TACE compared with others treated with curative methods were not compared. Further prospective studies including more cases and more variables could improve our results.

In conclusion, the radiological characteristics including complete lipidol retention and absence of residual tumor blush, had positive impacts on the radiological complete tumor response of patients with small-sized early-stage HCC receiving TACE.

## Author contributions

**Data curation:** Chieh-Ling Yen, Sheng-Shun Yang.

**Investigation:** Shou Wu Lee, Teng-Yu Lee, Chieh-Ling Yen.

**Methodology:** Shou Wu Lee, Teng-Yu Lee, Yu-Chi Cheng.

**Writing – original draft:** Yu-Chi Cheng, Sheng-Shun Yang.

**Writing – review & editing:** Shou Wu Lee, Teng-Yu Lee.
